# Local Antibiotic-Loadable Carriers for the Treatment of Chronic Osteomyelitis: A Narrative Review

**DOI:** 10.3390/bioengineering13040436

**Published:** 2026-04-08

**Authors:** Andrea Sambri, Alessandro Bruschi, Cristina Scollo, Massimiliano De Paolis

**Affiliations:** Orthopedic and Traumatology Unit, IRCCS Azienda Ospedaliero-Universitaria di Bologna, 40138 Bologna, Italy; alessandro.bruschi@aosp.bo.it (A.B.); cristina.scollo@aosp.bo.it (C.S.);

**Keywords:** chronic osteomyelitis, local antibiotics, biodegradable carriers, calcium sulfate, hydroxyapatite, bone substitute

## Abstract

Local antibiotic delivery has gained a central role as an adjunct to radical debridement in chronic osteomyelitis, allowing high antimicrobial concentrations at the infection site while reducing systemic toxicity. This narrative review summarizes the current clinical evidence on commercially available antibiotic-loadable bone substitutes, with particular focus on calcium sulfate (CaSO_4_)-based systems and biphasic calcium sulfate/hydroxyapatite (CaS/HA) composites. Nineteen studies were included. Differences in formulation, resorption kinetics, antibiotic elution profile and osteoconductive behavior are discussed, alongside clinical outcomes including recurrence of infection, reoperation rates and complication patterns. Finally, based on the currently available evidence and expert recommendations, practical guidance is proposed to support carrier selection in different clinical scenarios (cavitary vs. corticomedullary defects; high-risk soft tissue; polymicrobial or resistant infections). Across published series, although heterogeneous, infection eradication rates are generally high when local carriers are integrated into structured surgical protocols. Calcium sulfate carriers provide rapid resorption and robust early antibiotic release but are associated with higher rates of sterile wound drainage. In contrast, CaS/HA biocomposites demonstrate more gradual remodeling and radiographic integration, potentially improving defect consolidation and reducing wound-related morbidity, although leakage and cost considerations remain relevant.

## 1. Introduction

Chronic osteomyelitis (COM) is a devastating complication after trauma or orthopedic surgery [1], associated with a substantial clinical and socio-economic burden, often characterized by a relapsing course and prolonged disability.

It is characterized by low-grade inflammation, the presence of sequestrum (necrotic and dead bone) and fistulous tracts [2]. The devascularized nature of the sequestrum may protect bacteria from the host immune response, thus limiting the effectiveness of many antibiotics [3]. A critical component of COM pathophysiology is bacterial biofilm, which impairs immune clearance and can increase antibiotic tolerance [4]. Reported recurrence rates range from 10% to over 30%, depending on host status, anatomical site, and adequacy of surgical management. Even after apparently successful treatment, late recurrence may occur years after initial therapy, reflecting the persistence of dormant bacteria within biofilm or necrotic bone. These features underscore the importance of adopting standardized and aggressive treatment strategies.

The current gold standard for COM management is based on a combined surgical and medical approach, in which radical debridement of all infected and non-viable tissue represents the cornerstone of treatment. This is typically combined with systemic antibiotic therapy, ideally guided by microbiological cultures and administered for several weeks. In addition, appropriate dead-space management and skeletal stabilization, along with soft tissue reconstruction when required, are critical determinants of outcome. Multidisciplinary care involving orthopedic surgeons, infectious disease specialists, microbiologists, and plastic surgeons has been shown to significantly improve treatment success rates.

Despite well-established principles, treatment of COM remains complex and requires a dedicated multidisciplinary team [5,6]. It often needs multiple surgical interventions and a prolonged course of systemic antibiotic therapy [7,8]. Radical debridement of non-vital, infected, and fibrous tissue remains essential, often resulting in large bone and soft tissue defects [9,10,11,12]. Moreover, systemic antibiotic therapy is limited by toxicity thresholds and poor penetration into devascularized bone or cavities following debridement.

Local antibiotic delivery systems were introduced to address the limitations of systemic therapy and dead-space management. They can allow for a reduced number of surgical procedures, a shortened hospitalization period and fewer systemic side effects when compared to parenteral antibiotic therapy alone [13,14,15,16].

Early strategies relied on non-biodegradable carriers such as polymethylmethacrylate (PMMA) beads and spacers loaded with antibiotics. Their antibiotic release relies on surface diffusion, resulting in a burst followed by prolonged subtherapeutic release. This persistent low-dose exposure creates selection pressure for resistant organisms and may allow PMMA to become colonized and biofilm-covered once elution declines. Additionally, PMMA is non-biodegradable and often requires a second surgery for removal.

In contrast, biodegradable ceramics allow complete drug release within a finite time window and do not require extraction [17,18,19,20]. Local antibiotic carriers aim to combine several key functions within a single platform: delivery of high local concentrations of antibiotics reducing the risk of re-establishment of biofilm, support of bone regeneration, and avoidance of secondary removal procedures [21]. They can achieve concentrations 10–100 times the minimum inhibitory concentration (MIC), potentially approaching minimum biofilm eradication concentration (MBEC), while maintaining low serum levels.

Among the various materials investigated, calcium sulfate-based and biphasic calcium sulfate/hydroxyapatite biomaterials have gained particular prominence due to their biocompatibility, resorbability, and versatility as antibiotic carriers [22,23]. Calcium sulfate offers predictable resorption and rapid release of incorporated antibiotics, making it particularly suitable for early infection control [23]. However, rapid resorption may be associated with local wound complications and limited mechanical support [24,25]. The addition of hydroxyapatite to form biphasic composites aims to modulate resorption kinetics and provide a residual osteoconductive scaffold to support bone ingrowth and remodeling. These design principles reflect an effort to balance antimicrobial efficacy with biological integration and mechanical considerations.

Despite increasing clinical adoption, important questions remain regarding the optimal selection, handling, and indications of antibiotic-loaded bone substitutes in COM.

## 2. Methods

### 2.1. Study Design

This narrative review aims to provide an in-depth and comprehensive analysis of the literature on antibiotic-loaded biodegradable bone substitutes for the treatment of COM. The manuscript focuses on clinically available materials and the most frequently reported products in the literature, including calcium sulfate (CaS)-based carriers and biphasic calcium sulfate/hydroxyapatite (CaS/HA) biomaterials. A narrative approach was selected due to the substantial heterogeneity of published evidence (study design, populations, definition of osteomyelitis, surgical strategy, antibiotic protocols, and outcome reporting), which limits formal quantitative analysis. 

### 2.2. Literature Search Strategy

A comprehensive literature search was performed in PubMed, Scopus, and the Cochrane Library from database inception to December 2025. The search strategy combined controlled vocabulary (MeSH terms) and free-text keywords related to osteomyelitis and local antibiotic delivery systems. The search string was as follows: (“chronic osteomyelitis” OR “bone infection” OR “fracture-related infection”) AND (“local antibiotic” OR “antibiotic-loaded” OR “drug delivery”) AND (“calcium sulfate” OR “hydroxyapatite” OR “bone substitute” OR “biodegradable carrier” OR “ceramic”). Reference lists of included studies and relevant reviews were also manually screened to identify additional eligible articles.

All retrieved records were independently screened by two reviewers (AS, AB) based on title and abstract. Full texts of potentially eligible studies were then assessed for inclusion. Discrepancies were resolved through discussion and consensus.

### 2.3. Eligibility Criteria

The selection process followed PRISMA principles.

Studies were considered eligible if they met the following criteria:

1. Population: human subjects with COM of long bones and/or cavitary infected bone defects.
2. Intervention: use of biodegradable, antibiotic-loadable bone substitutes intended for bone defect filling and dead-space management. Materials without antibiotic-loading capability or lacking structural bone void filling properties were excluded.
3. Outcomes: report of at least one clinical outcome such as infection recurrence/eradication, wound complications, bone healing/remodeling, radiographic integration, reoperation rates, or adverse events.
4. Study type: randomized or non-randomized clinical trials, prospective or retrospective cohort studies, comparative studies, and larger case series (>5 patients).

Case reports and small cases series (<5 cases) were excluded; papers were excluded if purely technical notes without clinical outcomes, studies unrelated to bone infection (e.g., non-infected bone void filling), non-human preclinical studies, articles not available in full text and article in language other than English.

Specifically, biomaterials such as bioactive glass S53P4, which provide intrinsic antibacterial activity but are not antibiotic-loadable, and collagen-based antibiotic carriers (e.g., gentamicin–collagen fleeces), which do not provide structural bone support, were excluded to maintain a focused and clinically homogeneous analysis.

## 3. Results

The number of records identified, screened, and included is summarized in the PRISMA flow diagram.

**Figure 1:**
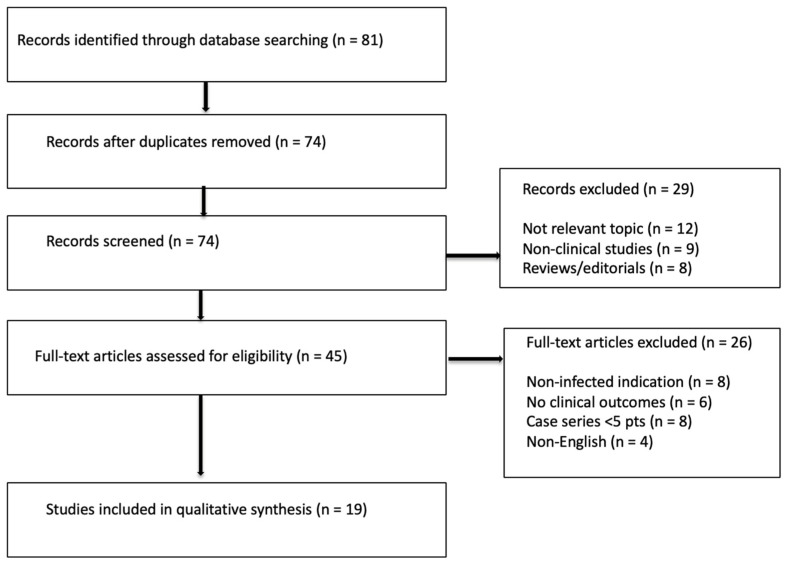
PRISMA flow diagram of the study selection process.

The initial search yielded 81 records. After removal of duplicates, 74 records were screened. Of these, 45 full-text articles were assessed for eligibility, and 19 studies were ultimately included in the review (Figure 1).

Antibiotic-loadable biodegradable bone substitutes have progressively replaced non-resorbable antibiotic-impregnated PMMA beads in the management of COM. Their combined role as local antimicrobial delivery vehicles and dead-space fillers has enabled “single-stage” treatment strategies, particularly in long bone infections, by delivering high local antibiotic concentrations while avoiding the need for later removal. However, despite their shared conceptual rationale, clinically used products differ substantially in biomaterial composition, antibiotic delivery profile, osseointegration capacity, and adverse-event pattern. These differences are clinically relevant because outcome measures in COM depend not only on antimicrobial effect (infection clearance) but also on biomechanical integrity, defect morphology, host factors, and the capacity for radiographic bone remodeling.

In the current clinical practice, four major platforms dominate the literature: calcium sulfate pellets preloaded with tobramycin (OsteoSet-T^®^), synthetic surgeon-mixed calcium sulfate beads (STIMULAN^®^), calcium sulfate/hydroxyapatite (CaS/HA) injectable composite preloaded with gentamicin or vancomycin (CERAMENT G/V^®^), and CaS/HA pellets designed to be intraoperatively loaded with multiple antibiotics (PerOssal^®^) (Table 1).

### 3.1. OsteoSet^®^/OsteoSet-T^®^

OsteoSet^®^ (Stryker, Kalamazoo, MI, USA) is a calcium sulfate (CaS) bone void filler that comes in pre-formed beads. OsteoSet-T^®^ is the commercial preloaded formulation containing 4% tobramycin in CaS pellets.

The largest clinical series is reported by Ferguson et al. [25] (195 COM es). In this cohort, recurrence occurred in 18/195 (9.2%) cases at mean of 10.3 months, with most recurrences within 2 years. Following additional surgery, infection was resolved in 97.9% (Table 2). Prolonged wound leakage was reported in 30/195 (15.4%) cases and was typically self-limited. Radiographic defect filling was often incomplete. Fracture of the treated segment occurred in 9/195 (4.6%) patients at mean of 1.9 years [25].

In the tibial-focused cohort by Humm et al. [24] (21 post-traumatic chronic tibial osteomyelitis), recurrence occurred in 1/21 (4.8%), and wound complications were common (52%) including early wound discharge in 7/21 (33.3%). One transient acute kidney injury was observed, highlighting the need to consider systemic exposure even with local carriers in susceptible hosts.

Additional evidence includes a retrospective series by Chang et al. [26] and McKee et al. [27], suggesting improved single-stage outcomes when OsteoSet-T^®^ was added to debridement compared with debridement alone.

### 3.2. CERAMENT™ G/V

CERAMENT™ (Bonesupport AB, Lund, Sweden) is a biphasic injectable bone substitute composed of calcium sulfate (≈60%) and hydroxyapatite (≈40%), commercially available preloaded with gentamicin (CERAMENT™ G) or vancomycin (CERAMENT™ V). It is designed to provide controlled resorption with an HA scaffold remaining after the calcium sulfate phase resorbs in 4–6 weeks.

CERAMENT is supported by one of the most extensively reported clinical datasets among antibiotic-loadable bone substitutes, largely originating from the Oxford Bone Infection Unit (Table 3).

McNally et al. [23] reported in a prospective series of 100 patients/105 bones that infection eradication was achieved in 96% after a single procedure at mean of 19.5 months, with few adverse events (fracture 3%, wound leakage 6%).

A more recent paper by the same group [28] confirmed encouraging results at mid- to long-term follow-up (mean 6 years) of the same patient cohort, with 94% of bones remaining infection-free.

A large radiographic/histological analysis in 163 COM cases [29] reported eradication in 95.7% after one stage procedure, and mean void filling of 73.8%. These data were confirmed histologically, with biopsies demonstrating active remodeling with osteoid, woven and lamellar bone formation.

Other cohorts showed more heterogeneous outcomes [30,31,32], potentially reflecting differences in defect type, host status, and soft tissue envelope. Niemann et al. [32] (20 patients) observed good functional recovery but high revision rates due to persistence or local wound complications (10/20 required revisions).

**Table 3:** Literature review of studies reporting on Cerament for the treatment of chronic osteomyelitis. NR: not reported. extended follow-up of the same cohort.

Study	Year	Type of Study	Sample Size	Sites of Infections	Follow-Up (Mean)	Recurrence of Infection Rate	Complications
McNally et al. [23]	2016	Prospective series	100 patients/105 bones	Long bones (multiple sites)	19.5 months (12 to 34)	4%	Wound leakage 6%; fractures 3%
McNally et al. [28]	2022	Prospective consecutive series	100 patients/105 bones	Long bones (multiple sites)	6.05 years (4.2–8.4)	6%	Wound leakage 6%; fractures 3%
Ferguson et al. [29]	2019	Retrospective analysis of consecutive series	163 cases	Long bones (multiple sites)	21.4 months (12–56);	4.3%	NR (study focused on imaging/histology)
Niemann et al. [32]	2022	Retrospective cohort	20	Multiple sites	17.5 months	10%	Wound leakage 5%
Drampalos et al. [30]	2020	Retrospective case series	15	Lower limb	14 months	13.3%	Wound leakage 1; delayed wound healing 2
Hoveidaei et al. [31]	2024	Case series	21	Multiple sites	10 months	4.8%	Delayed wound healing 1

### 3.3. PerOssal^®^

PerOssal^®^ (OSARTIS GmbH, Münster, Germany) is a resorbable ceramic bone substitute based on a calcium sulfate (CaS) matrix combined with nanocrystalline hydroxyapatite (HA), marketed as beads/pellets that can be loaded intraoperatively with antibiotics. The beads can be loaded with pathogen-directed antibiotics (often vancomycin, gentamicin, and/or rifampicin combinations depending on in vitro antibiotic susceptibility test) and are then packed into the cavity to deliver high local concentrations while the material gradually remodels (Table 4).

The mid-term Bologna experience reported by Sambri et al. [33] focused specifically on single-stage surgery with antibiotic-loaded PerOssal^®^ beads. This study suggests the feasibility of one-stage management, although interpretation is limited by its retrospective design and lack of a control group.

Radiographic analysis suggested progressive defect filling in many cases; however, the absence of standardized radiographic criteria limits interpretation.

Another retrospective clinical series [34] evaluated 97 patients treated for COM (minimum follow-up 24 months). The subgroup treated with curettage plus PerOssal^®^ (n = 52) achieved a healing rate of 86.5%, significantly higher than curettage alone. Among patients with recurrence, a delay in recurrence was observed; however, the clinical significance of this finding remains uncertain and may be influenced by study design and patient selection. Overall, while available studies suggest potential benefits of PerOssal^®^, the current evidence remains limited and heterogeneous, and conclusions should be interpreted with caution in the absence of high-quality comparative trials.

**Table 4:** Literature review of studies reporting on Perossal for the treatment of chronic osteomyelitis. NR: not reported.

Study	Year	Type of Study	Sample Size	Sites of Infections	Follow-Up (Mean)	Recurrence of Infection Rate	Complications
Romanò et al. [22]	2014	Retrospective comparative study (vs. bioactive glass S53P4 vs. Calcium sulphate)	27 Perossal (76 in total)	Long bones	22.1 months (range, 12–36)	11.1%	Reduced drainage vs. other calcium substitutes
Sambri et al. [33]	2023	Retrospective cohort	93	Femur 24; Tibia 52; other sites 17	≥12 months	8.6%	8% Leakage
Armbruster et al. [35]	2024	Retrospective cohort	82	Multiple sites	NR	19.5%	Revision 32.9%, mainly wound healing disorders and reinfections
Visani et al. [34]	2018	Retrospective cohort (vs. debridement alone)	52 Perossal (97 in total)	Long bones (multiple sites)	≥24 months	14%	NR

### 3.4. STIMULAN^®^

STIMULAN™ (Biocomposites, Ltd., Keele, UK) is a synthetic, high-purity calcium sulfate (CaS) carrier that can be formed intraoperatively as beads or a paste and loaded with antibiotics.

In clinical protocols, STIMULAN™ is mixed with antibiotics at the time of surgery and inserted into debrided bone cavities/medullary canals. Because the product does not contain preloaded antibiotics, selection is typically guided by culture and antibiotic sensitivity test, and the most commonly reported combinations include vancomycin with aminoglycosides (gentamicin/tobramycin) [20].

A recent randomized blinded clinical trial by Palo et al. [36] evaluated 95 patients with COM treated with surgical debridement and STIMULAN™ beads for local antibiotic delivery. (Table 5) Patients were randomized into three groups to compare antibiotic compositions and bead characteristics with recurrences observed in 2.1% of the cases. Other smaller series [37,38,39] reported higher rates of recurrent infections (7.7–23.3%). Zhou et al. [39] also reported high rates of Prolonged aseptic drainage (30.0%).

**Table 5:** Literature review of studies reporting on Stimulan for the treatment of chronic osteomyelitis. NR: not reported.

Study	Year	Type of Study	Sample Size	Sites of Infections	Follow-Up (Mean)	Recurrence of Infection	Complications
Palo et al. [36]	2024	Prospective multicenter randomized blinded study	95	Long bones	12 months	2.1%	NR
Zhou et al. [39]	2020	Retrospective study	42 patients	Tibia	42.8 months	11.6%	Prolonged aseptic drainage (30.0%)
Badie et. al. [37]	2018	Prospective protocol/cohort	30	Multiple sites	≥12 months	23.3%	Pathological fracture 1/30
Ferrando et al. [38]	2017	Retrospective comparative cohort (vs. Bioactive glass S53P4)	13 (25 in total)	Multiple sites	22 months (16–29)	7.7%	NR

## 4. Discussion

A key endpoint in COM surgery is recurrence of infection. Reported recurrence rates vary widely across the literature, reflecting heterogeneity in host status (Cierny–Mader class), extent of debridement, soft-tissue coverage, antibiotic choice/duration, and follow-up. Nevertheless, a few studies showed potential advantages in terms of infection control when local antibiotics were used in comparison to debridement without local antibiotics [22,26,34,38].

Moreover, despite heterogeneity among series, clinically relevant trends emerge when comparing carriers. OsteoSet-T^®^ has among the earliest and largest datasets [25,26]. These studies support the concept that calcium sulfate carriers can be effective adjuncts in infection eradication, particularly when combined with adequate surgical clearance. Other series using other carriers report a heterogeneous rate of re-infection, with variable recurrence rates depending on host factors, vascularity, and severity [40]. It must be highlighted that differences between products may be smaller than differences created by surgical protocols and host status, and that biomaterial choice must be integrated into a structured COM pathway including radical debridement and appropriate systemic antibiotic therapy.

Across biodegradable carriers, the most clinically relevant complication is wound leakage. Calcium sulfate resorption generates a transient ionic and osmotic microenvironment that can lead to sterile serous drainage. However, drainage rates appear higher in pure CaS carriers compared with CaS/HA composites. OsteoSet-T^®^ has particularly well-described drainage complications [25]. These findings highlight that rapid resorption can translate into clinically meaningful wound morbidity. STIMULAN^®^ series similarly report drainage as a key limitation. Palo et al. [36] analyzed forming and absorption time, suggesting that bead characteristics and absorption kinetics vary with antibiotic mixture, which may influence postoperative discharge risk. CERAMENT^®^ and Perossal appear associated with lower leakage rates than pure CaS. In the mid- to long-term follow-up of McNally’s prospective cohort, leakage from the wound was observed in only 6% [28]. The study by Niemann et al. reinforced that wound issues may occur but are generally manageable in standardized protocols and that outcomes are influenced by defect morphology and host factors [32]. The amount of leakage seems also to be particularly high in tibial infection with limited soft tissue coverage. Sambri et al. observed no leakage with the use of Perossal whenever an orthoplastic approach was used in tibial COM [33,41].

Rapidly resorbing CaS in large defects may theoretically compromise structural integrity if bone does not regenerate synchronously. Hydroxyapatite scaffold in biodegradable biphasic substitutes may integrate and remodel into host bone, reducing the risk of persistent cavitary defects [28]. Ferguson et al. [29] conducted a radiographic and histological analysis of CERAMENT G in COM, demonstrating progressive remodeling with evidence of new bone formation and incorporation over time. Radiographs suggested early resorption of the CaS fraction while HA persisted as osteoconductive framework, and histology confirmed osteoid deposition and remodeling rather than inert encapsulation. Similarly, Sambri et al. [33] documented mid-term radiological evolution after Perossal bead implantation, noting acceptable osseointegration and remodeling in the majority of treated cavities. These data support that nanocrystalline HA composites may favor progressive integration and defect filling, especially in cavitary lesions treated after radical debridement, thus potentially being more aggressive in the debridement.

Beyond the properties of the carrier itself, the interaction between the antibiotic and the biomaterial matrix plays a critical role in determining elution kinetics and antimicrobial efficacy. Antibiotic release is influenced by physicochemical characteristics such as molecular weight, solubility, and binding affinity to the carrier, as well as by the porosity and resorption profile of the material. Hydrophilic antibiotics such as aminoglycosides (e.g., gentamicin, tobramycin) typically demonstrate predictable release profiles from calcium sulfate-based carriers, whereas more complex molecules or combinations may exhibit variable elution behavior. In surgeon-mixed systems such as STIMULAN^®^ and PerOssal^®^, mixing protocols and antibiotic selection introduce an additional layer of variability. Factors such as antibiotic concentration, combination of multiple agents, and preparation technique can influence not only release kinetics but also the mechanical properties and resorption behavior of the carrier. Moreover, certain antibiotics may undergo degradation or reduced activity depending on local environmental conditions, including pH and temperature during setting. These considerations highlight that local antibiotic therapy is not solely dependent on the carrier but represents a combined biomaterial–drug system, where both components must be optimized to achieve effective infection control.

It should also be acknowledged that other clinically used materials, such as bioactive glass S53P4 and collagen-based antibiotic carriers, have demonstrated efficacy in the management of bone infections. However, these approaches differ conceptually from antibiotic-loadable bone substitutes, as they rely on intrinsic antibacterial properties or act as non-structural drug delivery systems. While they represent valuable adjuncts in specific clinical scenarios, their inclusion would have introduced heterogeneity in terms of mechanism of action and clinical application and was therefore beyond the scope of the present review.

Beyond currently available biodegradable carriers, recent advances have enabled the development of 3D- and 4D-printed scaffolds incorporating smart materials capable of responding to external or local stimuli such as light, pH, temperature, or magnetic fields. In particular, near-infrared (NIR)-responsive scaffolds have demonstrated the ability to trigger controlled drug release through photothermal mechanisms, allowing non-invasive and temporally precise modulation of therapeutic delivery. A recent study [42] described a dual-drug-loaded 3D-printed scaffold capable of sequential and on-demand release, combining early chemotactic signaling with later osteogenic stimulation, thereby aligning drug delivery with the physiological phases of bone healing. Furthermore, 4D-printed “deployable” scaffolds can undergo shape adaptation and functional changes over time, enabling improved defect filling and dynamic drug release in response to environmental cues. Although these approaches remain largely preclinical, they represent a promising direction toward “smart” implants capable not only of delivering antibiotics but also of sensing infection and responding dynamically, potentially improving infection control while minimizing unnecessary antibiotic exposure.

This narrative review has several limitations that must be acknowledged. First, the available clinical evidence on antibiotic-loadable biodegradable bone substitutes remains heterogeneous in terms of patient populations, infection definitions, anatomical sites, extent of bone defects, and host status, limiting the possibility of direct comparison among different products. Second, most clinical data arise from retrospective single-center case series (even small in sample size), often without control groups, and are therefore affected by selection bias, incomplete reporting of complications, and variability in follow-up duration; importantly, late recurrences may be underestimated in studies with shorter observation periods. Third, definitions of treatment success in bone infection are not uniform across the literature. Even though Consensus definitions proposed by the Musculoskeletal Infection Society and subsequent International Consensus Meetings emphasize that treatment success should incorporate multiple criteria, including absence of clinical and microbiological evidence of infection, no requirement for further surgical intervention, and no need for long-term suppressive antibiotic therapy [43], outcomes such as “infection eradication” are inconsistently defined across reports. Many studies included in this review define success solely as absence of recurrence at follow-up, without accounting for ongoing antibiotic use or subclinical infection. This variability limits direct comparison between studies and may contribute to the wide range of reported outcomes. Standardization of outcome reporting remains essential for improving comparability and guiding clinical decision-making. It should also be acknowledged that some of the studies included in the PerOssal^®^ section originate from the authors’ institution. Although all relevant studies meeting inclusion criteria were considered, this may represent a potential source of selection bias. Moreover, part of the evidence supporting CaS/HA composites derives from longitudinal follow-up studies of the same patient cohort, rather than independent populations, which should be considered when interpreting the apparent consistency of outcomes. In addition, a formal quality assessment of included studies using standardized tools was not performed due to the narrative design and heterogeneity of the available evidence. Finally, antibiotic choice and dosing are frequently surgeon-dependent (particularly for surgeon-mixed systems), and mixing protocols are not uniformly reported, limiting reproducibility and raising regulatory considerations.

## 5. Conclusions

Carrier selection should be individualized based on defect type (cavitary vs. segmental), need for mechanical stability, soft tissue quality, need for scaffold persistence, microbiology and required duration of antibiotic elution.

Calcium sulfate systems provide strong burst elution and complete biodegradation but have drainage risk, whereas biphasic CaS/HA materials provide osteoconductive scaffold persistence and more gradual remodeling. In cavitary defects with robust soft tissues, pure CaS systems (STIMULAN or OsteoSet-T) may be useful for rapid antibiotic delivery and complete resorption. However, in tibial infections, large dead space, or settings with tenuous wound healing, drainage rates can be clinically significant, favoring biphasic CaS/HA composites.

Cerament may be particularly advantageous in corticomedullary defects, defects requiring longer scaffold presence, and scenarios where radiographic remodeling is desired and leakage risk should be minimized. Its standardized preloaded antibiotic formulation improves reproducibility. PerOssal may represent a useful option when flexibility in antibiotic choice is needed (including rifampicin combinations), while maintaining HA-driven osteoconductivity. It may therefore be favored in polymicrobial infections or resistant pathogens requiring tailored local antibiotic regimens.

Overall, these bone substitutes share the ability to support high infection eradication when combined with radical debridement; nevertheless, their differences in resorption kinetics, scaffold persistence, and drainage risk justify individualized selection according to clinical scenario rather than infection clearance alone.

## Figures and Tables

**Table 1:** Characteristics of antibiotic-loadable bone substitutes currently available on the market. Elution profiles vary substantially depending on antibiotic type, concentration, carrier preparation, and experimental conditions; reported values are derived from heterogeneous in vitro and in vivo studies and should be interpreted as approximate ranges rather than fixed durations.

Product	Material/Formulation	Configuration	Antibiotics (Preloaded)	Antibiotics (Mixable/Loadable)	Reported Elution Time (>MIC)	Osteoconductivity	Leakage
OsteoSet-T^®^	Calcium sulphate (CaSO4) hemihydrate	Pellets/beads (e.g., 4.8 mm × 3.3 mm)	Tobramycin 4%	—	in vitro bactericidal to day 22; in vivo therapeutic local levels to day 28	Limited	High
CERAMENT-G^®^/CERAMENT-V^®^	60% calcium sulphate + 40% hydroxyapatite	Injectable paste	Gentamicin 17.5 mg/mL (G); Vancomycin 66 mg/mL (V)	—	28 days	High	Low
PerOssal^®^	48.5% calcium sulphate + 51.5% nanocrystalline hydroxyapatite	Pellets (6 mm)	—	Vancomycin; Gentamicin; Tobramycin; Rifampicin	Variable (≈7–14 days depending on antibiotic and formulation)	High	Moderate
STIMULAN^®^	Calcium sulphate hemihydrate	Beads (3, 4.8, 6 mm); paste; bullets (7–9 mm)	—	Vancomycin; Gentamicin; Tobramycin	Variable; sustained release reported up to several weeks depending on antibiotic and mixing protocol	Low	High

**Table 2:** Literature review of studies reporting on OsteoSet for the treatment of chronic osteomyelitis. NR: not reported.

Study	Year	Type of Study	Sample Size	Sites of Infection	Follow-Up (Mean)	Recurrence Rate	Complications
Ferguson et al. [25]	2014	Retrospective case series	195	Long bone (multiple sites)	NR (recurrence mean 10.3 mo)	9.2%	Early leakage 36/195 (18.5%); prolonged leakage 30/195 (15.4%); 3/46 (6.5%); fractures reported
Humm et al. [24]	2014	Retrospective review	21	Tibia	15 months	4.8%	Wound complications 52%; wound discharge/leakage 7/21 (33%)
Chang et al. [26]	2007	Retrospective comparative cohort (vs. debridement alone)	25 OsteoSet-T (65 total)	Femur (26), Tibia (32), Humerus (5), Radius/Ulna (2)	75 months	OsteoSet-T: 20%; Debridement only: 40%	NR
McKee et al. [27]	2002	Prospective consecutive trial	25	Tibia (15), Femur (6), Ulna (3), Humerus (1)	28 months (range 20–38)	8%	Fracture 3/25 (12%); sterile draining sinuses 8/25 (32%)
Gitelis et al. [21]	2002	Case series/early clinical experience	6	Multiple sites	2.3 years (range 1.5–3.3)	0%	No significant drainage; no fractures.

## Data Availability

No new data were created or analyzed in this study. Data sharing is not applicable to this article.
